# Urban Individuals of Three Rove Beetle Species Are Not More Exploratory or Risk-Taking Than Rural Conspecifics

**DOI:** 10.3390/insects13080757

**Published:** 2022-08-22

**Authors:** Tibor Magura, Roland Horváth, Szabolcs Mizser, Mária Tóth, Dávid D. Nagy, Réka Csicsek, Emőke Balla, Gábor L. Lövei

**Affiliations:** 1 Department of Ecology, Faculty of Science and Technology, University of Debrecen, Egyetem sq. 1, H-4032 Debrecen, Hungary; 2 ELKH-DE Anthropocene Ecology Research Group, University of Debrecen, Egyetem sq. 1, H-4032 Debrecen, Hungary; 3 Department of Agroecology, Research Centre Flakkebjerg, Aarhus University, DK-4200 Slagelse, Denmark

**Keywords:** staphylinids, urbanization, exploratory behavior, risk-taking behavior, human disturbance

## Abstract

**Simple Summary:**

Urbanization-derived disturbances and threats, as well as changes in environmental and habitat parameters act as selection pressures on various features of urban-dwelling animals, including their behavior. Earlier studies on vertebrates showed that urban individuals are more exploratory and bolder than their rural counterparts. Similar analyses on invertebrates are rare, therefore we studied the exploratory and risk-taking behavior of individuals of three rove beetle species from rural and urban populations during their main reproductive period. Beetles of all three studied species responded consistently in the different behavioral tests. The exploratory behavior of beetles was consistent over time indicating the existence of personalities, but did not differ in differently urbanized habitats. *Ocypus nitens* males, however, were significantly more exploratory than females which can be explained by the active searching of males for mating partners.

**Abstract:**

Urbanization is creating changes in environmental and habitat conditions, as well as creating disturbance and threats to urban-associated species. Some traits, such as high exploratory and risk-taking behavior, are beneficial to allow colonization of urban habitats and coping with urbanization-derived pressures. In this study the exploratory and risk-taking behavior of rural and urban individuals of three forest-associated rove beetle species were tested during their main reproductive period by five frequently used behavioral measures. Individuals of all studied species were similarly ranked by all behavioral measures, indicating that the studied rove beetles responded consistently in the different contexts. However, the behavior of beetles was consistent over time for all/most studied species only by using two measures of exploratory behavior. These provide evidence for the existence of the exploratory dimension of personality in rove beetles. We found a higher exploratory behavior in males than females in *Ocypus nitens* which can be explained by the active searching of males for mating partners. There were no urbanization-related differences in the exploratory behavior of individuals, suggesting that behavioral changes (being more exploratory) may not yield additional fitness benefits in these rove beetle species with good dispersal capacity.

## 1. Introduction

Human activities, including agriculture, forestry, and urbanization are major and rapidly growing components of global change, causing considerable biodiversity loss [1]. Of these, urbanization is a process whereby more and more habitat is being brought under urban land use, accompanied by a surge in urban population growth and the spread of urban lifestyle [2]. Nowadays, about 55% of the human population lives in and around cities and this proportion will increase in the decades ahead [3,4]. In urban areas, the remaining natural habitat fragments are often isolated, limiting species and nutrient flows between habitat patches [5,6]. Urbanization also considerably alters pollutant deposition [7], climatic parameters [8,9], nutrient availability [10], and a range of biological processes such as decomposition [11], mineralization [12], gene flow [13], and community composition [14,15].

Urbanization is a key element of rapid human-induced environmental change [16,17]. Processes associated with urbanization cause changes not only in the structure and composition of habitats but also in their environmental parameters, which induce various stress effects on living organisms, modifying their activity patterns, spatial distribution, phenology, condition, productivity, behavior, and biotic interactions [18,19,20]. Despite this, cities also play an extremely important role in maintaining biodiversity, as they are generally located near biodiversity hotspots [21], and urban habitats still contain members of many groups of organisms (e.g., 30% of birds and 5% of plants of the global diversity), including both endemic and threatened species [22,23]. This is why urban diversity is particularly important, and to maximize the economic (e.g., ecosystem services) and environmental benefits that cities provide, we need to understand how different species can adapt to this new environment and how the negative impacts of the urban environment can be mitigated [24]. To do this, the impacts of urbanization need to be investigated at different levels of biological organization, from populations to communities [25]. Such studies have so far been carried out mainly on vertebrates (mammals [25,26] and birds [27,28]) and plants [29,30]. There are a lot fewer studies on terrestrial arthropods (but see [31,32]); nonetheless, the available data suggest that urbanization is a global threat to insect diversity [33]. Among the terrestrial arthropods, rove beetles (Coleoptera: Staphylinidae) are suitable for urbanization studies [34,35] due to their species richness, abundance, ecological variability [36], and the availability of simple sampling methods [37]. In addition, urbanization has a considerable impact on rove beetles in urban habitats at different levels of biological organization [35,38].

Living organisms can respond to changes in their environment at different levels. The fastest reactions to changes in environmental conditions are at the level of individual behavior. However, behavior is much more complex than a set of reflexive responses to triggers, and some individual animals respond consistently to environmental conditions experienced throughout their lives, indicating individual-specific personality [39,40]. The interpretation of such personality as adaptive plasticity is currently poorly understood. The specific environmental conditions created by urbanization in urban habitats put pressure on the animals living there, leading to selection for certain traits [41,42]. Species with a wide range of tolerance (habitat generalists [43,44]) or species that increase their tolerance to the conditions created by urbanization [45] have a considerable advantage. Certain behavioral traits, such as high exploratory behavior and high risk-taking, are advantageous for coping with and/or colonizing habitats featuring urbanization-induced changes [46,47]. Previous studies on vertebrates provide evidence that individuals in urban habitats are more exploratory and bolder than their rural counterparts [48,49]. Therefore, we hypothesized that rove beetles occurring in urban habitats would also display more exploratory and risk-taking behavior than conspecific individuals in rural habitats.

## 2. Material and Methods

### 2.1. Study Area and Sampling Design

In this study we collected rove beetles from the two extremes of the rural–urban gradient, in and around the city of Debrecen (Eastern Hungary). We selected eight forested sampling sites along the gradient (4 rural and 4 urban sites). Rural sites were situated in a continuous forest with size of 1082 ha, in old (>130 years) forest stands dominated by English oak (*Quercus robur*). All selected urban sites were in fragments of the same once-continuous English oak dominated old forest. The criteria for classifying sampling areas as rural or urban was the ratio of the built-up area vs. the natural habitats in a 1000 m radius around the studied sites measured by the ArcMap software using aerial photographs. In the urban area, the built-up part exceeded 60%, while in the rural one, there were no buildings [50]. Each site was at least 3 ha and at least 250 m from each other. In the urban forest patches, the larger fallen branches and trunks were shredded and left on the ground, while the shrub layer was heavily thinned. The paths were paved, and the human disturbance was considerable. In contrast, there was no regular forest management in the rural forest stands, and the human pressure was minimal.

We collected rove beetles during their main activity periods using live pitfall traps. We emptied the traps twice a week from the beginning of April to the end of June 2020. There were 15 live-capture pitfall traps without preservatives at each site (2 areas × 4 sites × 15 traps per site = 120 traps in total). We placed the traps randomly at least 10 m apart from each other. In order to avoid edge effect, all traps were at least 50 m from the nearest forest edge [51]. The traps were made of plastic containers (170 mm long × 110 mm wide × 105 mm deep) with crumbled leaves placed inside to allow the capture to hide and to prevent predation by other large arthropods. We covered the traps with fiberboard lids (20 × 20 cm) to protect them from litter, rain, and vertebrate predators. We delivered the collected rove beetles to the laboratory, and identified them to species level and sexed using standard keys [52]. We placed each individual in a Petri dish (90 mm diameter) with wet filter paper. Only water was available for the beetles because dehydration could influence their behavior and movements.

### 2.2. Test Organisms

Behavioral traits were measured on three common species of rove beetles. *Abemus chloropterus* (Panzer, 1796) is a medium sized (9.5–12.5 mm), endangered predatory species in Central-Europe, inhabiting mainly forests [53]. In the studied region (Great Hungarian Plain) this very rare forest specialist species with good dispersal power (macropterous) is active from April to July [54]. *Ocypus nitens* (Schrank, 1781) is a large (12–20 mm), common predatory rove beetle with good dispersal power via flying. In the studied region this species is abundant mainly in forested habitats [37] with an activity period from February to December [54]. *Platydracus fulvipes* (Scopoli, 1763) is a relatively large (13–19 mm), widespread predatory species of moist deciduous forests, with limited flight capacity. Its activity period lasts from May to August [54].

### 2.3. Testing, Measuring, and Evaluating Behavioral Parameters

After transportation to the laboratory, beetles were kept under standardized laboratory conditions (24 °C, 40% relative humidity and natural L:D cycle), and allowed to rest for 2 h, during which they had access to water but no food. After this resting period, beetles were tested individually. First, we measured their activity in a new environment, also known as the “open-field” test [55,56], which is often used to assess exploratory behavior [46,57,58]. The test environment constituted of an open, white plastic box (364 × 230 mm), the bottom of which was divided into 35 equally sized squares. The individual was placed in the middle of the box and covered it with a 55 mm diameter Petri dish. When the beetle finished moving, the lid was lifted and the beetle’s movements recorded for 90 s with a GoPro HERO6 camera (CHDHX-601-FW). We recorded the number of squares covered by individuals (henceforth referred to as no. squares visited), the number of squares not adjacent to the wall that were entered (henceforth referred to as no. inner squares visited) and the time until the individual reached the wall of the plastic box (henceforth referred to as time to wall). The number of squares visited and time to wall are recognized measures activity and exploration in arthropods [46,58,59,60], while the inner square visit is considered a parameter of risk-taking/boldness [58]. Immediately after testing the new environment, we investigated the response to threats. The escape response of individuals to a disturbance was tested in a ring-shaped arena divided into eight equal segments [58]. We placed rove beetles individually in the arena and waited until they stopped moving. Escape behavior was then induced by a mechanical stimulus: the back of the rove beetle was gently tapped with a small forceps. We recorded the time spent running (escape duration) and the number of segments crossed (escape distance). The test ended when the beetle stopped moving. We regarded escape duration and distance as a response to threat [58]. The arena was cleaned with 70% ethanol after every tenth test, or if the individual defecated. To assess the repeatability of behavior, we performed the tests twice on each individual, with 24 h between the two occasions. This interval is sufficient to estimate the repeatability of the measured behavioral variables [61]. Furthermore, testing individuals only twice prevented them from becoming habituated to the experimental conditions [58,61].

### 2.4. Statistical Analyses

We investigated the impact of urbanization level (non-urbanized vs. urbanized) on the measured parameters using generalized linear mixed models (GLMM) with the *lme4* package [62]. We tested the best-fitting probability distribution for our response variable using the *car* [63] and *MASS* [64] packages. Based on the results, we modelled the response variables with count data (no. squares visited, no. inner squares visited, and escape distance) with a Poisson distribution using a log-link function, while for the other variables (time to wall and escape duration) we used a normal error distribution with the log-link function [65]. Fixed effects were the level of urbanization, sex of the studied individual and their interaction. In the models we also took into account the nested design of our sampling (sampling sites were nested within sampling areas). In the models on behavioral measures, we considered trials as repeated measures and added the observer as random factor. When GLMM showed a significant difference between the means, we used the LSD test for multiple comparisons between means [65].

To study whether the behavior of rove beetles was consistent across contexts, we calculated Kendall’s coefficient of concordance including all behavioral measures (mean values of the two experiments) using the *DescTools* package [66]. We computed Spearman’s rank correlations to assess consistency of individuals’ behavioral measures between the experiments using the *RVAideMemoire* package [67], and we estimated the repeated probabilities from the GLMMs with the individuals IDs as a random term using the *rptR* package [68]. We performed an agglomerative cluster analysis to detect possible correlations between the different behavioral measures determining personality dimensions [58,60,69]. We calculated a dissimilarity matrix for the behavioral measures (mean values of the two tests) using Spearman’s rank correlations (one minus the absolute value of the correlation coefficients). Thereafter, we performed an agglomerative clustering with the Ward fusion method using the *cluster* package [70]. We identified personality dimensions (clusters of correlated behavioral measures) by investigating the overall average silhouette width values for the given number of clusters [71].

## 3. Results

In the sampling period (from April to June 2020) 233 individuals of the three studied rove beetle species were captured and tested (Table 1). We collected 99 individuals (28 females and 71 males) from the rural sites, and 134 beetles (23 females and 111 males) from the urban ones (Table 1). There were few urban females caught of *O*. *nitens* and *P*. *fulvipes* (Table 1).

**Figure 1:**
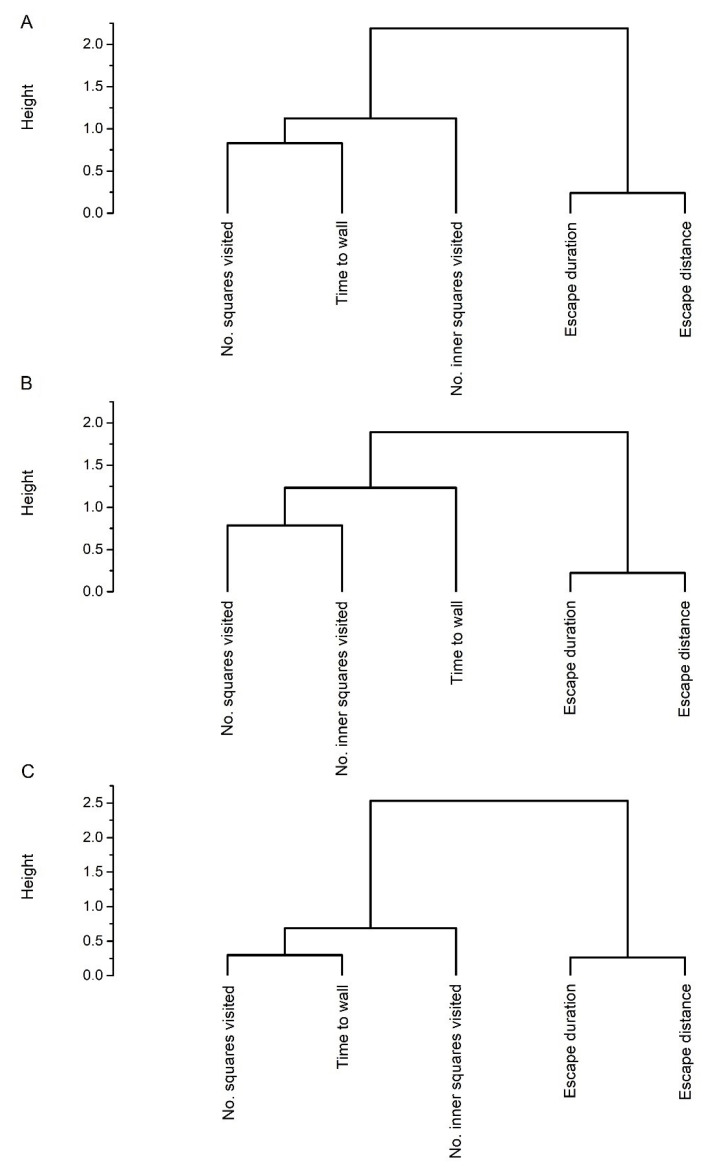
Grouping of the studied behavioral measures by agglomerative cluster analysis (agglomerative coefficient: 0.70, 0.66, and 0.86, respectively) for *Abemus chloropterus* (**A**), *Ocypus nitens* (**B**), and *Platydracus fulvipes* (**C**).

The Kendall’s coefficient of concordance was significant for all three species (*A*. *chloropterus:* W = 0.5992, χ^2^ = 182.14, df = 4, *p* < 0.0001, *O*. *nitens:* W = 0.5481, χ^2^ = 162.23, df = 4, *p* < 0.0001, *P*. *fulvipes:* W = 0.5762, χ^2^ = 191.29, df = 4, *p* < 0.0001), meaning that beetles were similarly ranked by all behavioral measures. These results indicate that the studied rove beetles responded consistently in the different behavioral tests. The number of squares visited was significantly rank-consistent and/or was significantly repeatable between the two consecutive trials for all three species (Table 2). The behavior of rove beetles measured by the number of inner squares visited, however, was not consistent between the trials (Table 2). Furthermore, the time to wall was significantly rank-consistent and repeatable for *A*. *chloropterus* and *P*. *fulvipes*, while escape duration and distance was consistent over time only for *O*. *nitens* (Table 2). The studied behavioral measures could be divided into two groups for all studied rove beetle species by agglomerative cluster analysis using the Spearman rank correlations (Figure 1). These clusterings were confirmed by the assessment of the average overall silhouette widths (Figure S1). The number of squares visited, the number of inner squares visited, and the time to wall were clustered into the first group, representing the exploratory dimension of beetles’ personality. The escape duration and the escape distance formed the second group, which can be considered as the risk-taking dimension of the beetles’ personality (Figure 1).

For all three rove beetle species, the correlation between the number of squares visited and the number of inner squares visited was positive and consistently significant. The relationship between the number of squares visited and the time to wall was also always significant, but negative. The escape duration and the escape distance were significantly positively correlated in all studied species (Table S1).

**Figure 2:**
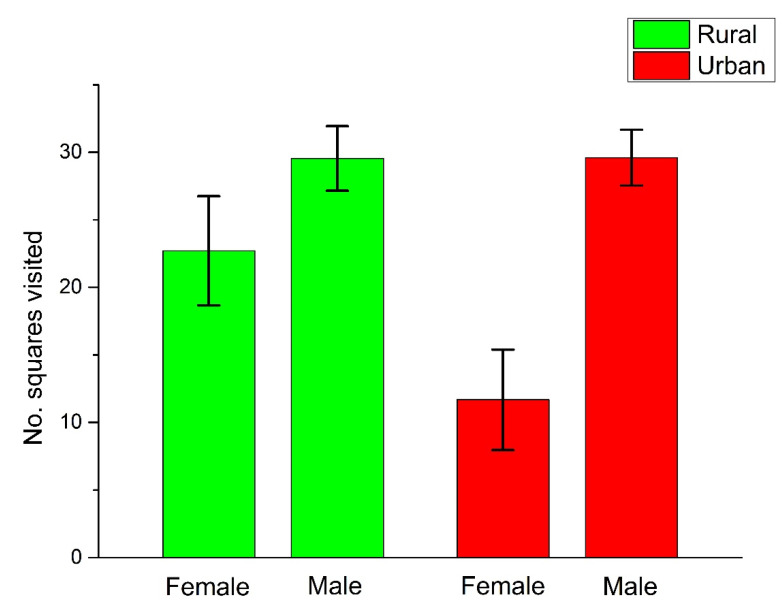
Mean (±SE) values of the number of squares visited by rural and urban *Ocypus nitens* individuals.

Of the behavioral measures of the exploratory dimension, the number of squares visited by *O*. *nitens* males was significantly higher than that of females, but other sexual differences in the behavioral measures were not significant (Table 3, Figure 2). Neither the urbanization level, nor the interaction between the urbanization and sex were significant factors explaining the number of squares visited, the number of inner squares visited, and the time to wall parameter (Table 3 and Table S2). Regarding the risk-taking personality dimension, urbanization level, sex, and their interaction were not significant explanatory factors on the escape duration and distance (Table 3 and Table S2).

## 4. Discussion

### 4.1. Behavioral Measures and Personality

In behavioral ecology, personality is defined as the behavioral differences among individuals of the same species, which are consistent over time and across different situations or contexts [72,73,74]. Behavioral traits that represent personality are often intercorrelated, clustered together and referred to as “behavioral syndromes” [72,73]. Behavioral traits (e.g., high exploratory behavior and high risk-taking) are usually described using standardized behavioral measures in behavioral tests under standardized laboratory conditions or on the field [72]. Most personality studies have been conducted on vertebrates; studies on invertebrates are few, even though the taxonomic diversity of invertebrates is much bigger than that of vertebrates [74].

In our study, the number of squares visited was significantly consistent between the trials in all three rove beetle species, while the number of inner squares visited was not. The number of zones crossed/visited by individuals in a new environment (“open-field” test) is often used to assess activity and exploratory behavior. Similarly to our results with rove beetles, the number of zones crossed/visited by ground beetles was significantly consistent [46,58,75]. However, contrary to our results, the number of inner zones crossed/visited by other beetles was also significantly [58,59,72] or marginally significantly [76] consistent over time. The number of inner zones visited is a parameter of boldness [58,75]. Therefore, interspecific variance in the inner zone visit can be explained by the difference in boldness between beetle species. In the studies where inner zone visits were significantly consistent over time, less bold beetle species (ground beetles [19,58] or leaf beetles [59,76]) were most likely to consistently respond to a novel environment. In contrast, the behavior of bolder species, such as rove beetles [77], seems unpredictable, as it is not possible to consistently predict whether they will escape or react aggressively when facing a novel situation. Based on the above, the number of inner zones crossed/visited by individuals is not suitable when testing beetles with confirmed aggressive behavior.

The time to reach the wall response was consistent for two species in our experiments. Similarly, this parameter is significantly consistent between trials (but see [78]) for firebugs [60], leaf beetles [59,76], and ground beetles [75], and this seems a reliable measure of exploratory behavior in arthropods [46,58,59,60].

In our study, measures of escape behavior after a simulated attack by a mechanical provocation was consistent over time only in one of the studied rove beetle species. However, earlier experiments on other beetles [58,75] indicated consistency. This difference can also be explained by differences in boldness. The reaction of bolder species, such as rove beetles, to a mechanical stimulus (escape or attack) is unpredictable, probably contributing to the non-consistency between trials. Indeed, several individuals in our experiments turned towards the stimulus-inducing forceps and attacked it. Based on these results, using simulated attacks to measure escape behavior is not recommended for beetles with confirmed aggressive behavior.

The activity and explorative behavior of rove beetles can be reliably assessed by tracking their movements, and counting the number of zones crossed, as well as measuring the time when the individual reached the wall of an experimental arena. Using these measures, we showed that rove beetle individuals behaved consistently over time. It is the first time that behavioral reaction by rove beetles was examined to uncover the presence of personality in these beetles. In addition, we demonstrated that the number of squares visited and the time to wall clustered together for all three studied species, possibly representing the exploratory dimension of their personality. Only one earlier study on a carabid beetle [75] measured simultaneously the number of zones crossed and the time to reach the wall of the arena, also indicating their relatedness.

### 4.2. Sex-Specific Differences in Behavior

Males of *O*. *nitens* visited significantly more squares of the arena than the females. The number of zones/sectors crossed/visited in a novel environment was not significantly different between sexes of either a ground beetle, *Carabus convexus* [72] or a tenebrionid, *Tenebrio molitor* [79]. However, Schuett et al. [46], testing the exploratory behavior in a novel environment of four ground beetle species (*Abax parallelepipedus*, *Carabus nemoralis*, *Nebria brevicollis,* and *Pterostichus oblongopunctatus*), showed that in three species, males visited more squares than females. Males of ground-dwelling beetles are generally more active than females, especially during the breeding period, when they are searching for mating partners [80]. Higher trapping rates of male than female rove beetles (see Table 1) from April to June (in the main reproductive period of the studied rove beetle species) also support the greater mobility of males compared to females, possibly for the same reason.

### 4.3. Urbanization and Behavioral Measures

Urbanization-driven alterations in environmental parameters, as well as anthropogenic disturbance (e.g., presence of humans, traffic, light and noise pollution) trigger well-documented changes in the behavior of urban birds [81,82,83,84] and mammals [49,85]. Contrary to the above studies on vertebrates, our results showed that the urbanization level (rural vs. urban) was not a significant factor explaining the exploratory and/or risk-taking behavior in rove beetles. Unfortunately, studies of urbanization-associated behavior changes on invertebrates are very scarce. In adult butterflies, tested under common garden conditions, habitat type (woodland, agricultural, or urban habitat) had no significant effect on their activity or boldness [47]. In the city of Hamburg, individuals from more urbanized sites of three ground beetle species (*C. nemoralis, N. brevicollis,* and *P. oblongopunctatus*) showed more square visits in a test arena than those from less urbanized sites, but only in the first of a two-year study [46]. In another study, the number of visited squares was marginally different between rural and urban ground beetle individuals, but risk-taking was not [75]. However, two other measures related to the exploratory dimension of beetle personality were significantly higher for rural than urban beetles [75]. Our non-significant results and the previous contradictory findings may raise concerns about whether urbanization uniformly triggers an increase in exploratory behavior in beetles. It is plausible to assume that higher mobility will result in less isolation between rural and urban populations, and if such between-habitat mobility is frequent enough, no selected responses would emerge. For example, adults of the carabid *N*. *brevicollis* are mobile enough to immigrate from their urban habitat to nearby suburban ones in Denmark [86]. Transplantation experiments could shed light to this possibility, where rural individuals are transplanted to urban ones. However, in-field behavioral tests have logistical and standardization (e.g., similar temperature conditions) challenges.

Urbanization-related environmental changes and disturbances have a documented negative impact on rove beetles [38]. In fact, the abundance of the three studied, forest-associated hygrophilous rove beetle species significantly decreased in the studied urban habitats [37]. However, body condition (expressed by fresh body mass) was not significantly different between rural and urban individuals of the same sex (results not shown). Despite the negative influence on abundance, our findings showed no significant difference in exploratory and risk-taking behavior. The studied rove beetle species are highly mobile, since *A. chloropterus* and *O. nitens* have a good flying ability, while *P. fulvipes* has a good walking ability [54]. It seems that their good dispersal ability allows individuals to find suitable microhabitats (e.g., for feeding and breeding) even in urban habitats, so an enhanced exploratory behavior may not yield further fitness benefits. Furthermore, these beetles are the top predators of the ground-dwelling consumer and decomposer guild, often aggressive and risk-taking, which could explain the lack of an increased boldness/risk-taking personality dimension in urban individuals.

## 5. Conclusions

We found that the activity and exploratory behavior of rove beetles can be reliably and consistently assessed in a test arena by counting the number of equally sized zones of the arena crossed by beetles, as well as by measuring the time when the individuals reached the wall of the arena. Using these behavioral measures we showed the existence of personalities in individuals from wild populations of three forest-associated hygrophilous rove beetle species. Sex-related differences in the exploratory behavior of *O*. *nitens* could be explained by the generally higher activity of males, especially during the breeding period, when they are actively searching for mating partners. Urbanization level (rural vs. urban), however, had no significant effect on the exploratory behavior of these rove beetles, possibly because their good dispersal ability allows them to easily find suitable microhabitats even in their altered urban habitats, so behavioral changes (being more exploratory) would not deliver additional fitness benefits.

## Figures and Tables

**Table 1:** Number of the sampled rove beetle individuals in rural and urban habitats, April—June 2020.

Species	No. of Rural		No. of Urban		Total
	Females	Males	Females	Males	
*Abemus chloropterus*	6	19	15	36	76
*Ocypus nitens*	12	20	3	39	74
*Platydracus fulvipes*	10	32	5	36	83

**Table 2:** Spearman rank-correlation (RS) and (adjusted) repeatability (r) for the behavioral measures of the two consecutive trials. Values in bold denote significant (*p* < 0.05) consistencies.

Rove Beetle Species	Behavioral Variable	Spearman’s Rank-Correlation RS (95% CI) *	Repeatability r (95% CI) *
*Abemus chloropterus*	No. squares visited	**0.3106 (0.0894; 0.4977)**	0.183 (0; 0.382)
	No. inner squares visited	0.0968 (−0.1120; 0.3113)	0 (0; 0.2)
	Time to wall (sec)	**0.3380 (0.0827; 0.5497)**	**0.191 (0; 0.396)**
	Escape duration (sec)	0.1245 (−0.1161; 0.3519)	0 (0; 0.208)
	Escape distance (no. segments)	0.1765 (−0.0572; 0.3773)	0.046 (0; 0.254)
*Ocypus nitens*	No. squares visited	**0.4469 (0.2189; 0.6160)**	**0.332 (0.114; 0.529)**
	No. inner squares visited	0.1034 (−0.1202; 0.3274)	0.067 (0; 0.243]
	Time to wall (sec)	0.1194 (−0.1102; 0.3299)	0 (0; 0.23)
	Escape duration (sec)	**0.2304 (0.0051; 0.4483)**	0.115 (0; 0.375)
	Escape distance (no. segments)	**0.3439 (0.1050; 0.5566)**	**0.399 (0.113; 0.566)**
*Platydracus fulvipes*	No. squares visited	**0.4995 (0.2992; 0.6591)**	**0.446 (0.221; 0.622)**
	No. inner squares visited	0.1604 (−0.0620; 0.3718)	0.143 (0; 0.317)
	Time to wall (sec)	**0.3918 (0.1651; 0.5879)**	**0.247 (0.079; 0.444)**
	Escape duration (sec)	0.1606 (−0.0492; 0.3618)	0 (0; 0.209)
	Escape distance (no. segments)	0.0838 (−0.1313; 0.3166)	0.051 (0; 0.224)

* Confidence intervals (CI) was calculated using 1000 bootstraps.

**Table 3:** Summary of GLMM results and post hoc tests on behavioral measures of the three studied rove beetle species in differently urbanized (non-urbanized vs. urbanized) forested habitats (*p*-values in bold denote significant effects).

Response Variable	Fixed Effect	Estimate ± SE	χ^2^	df	*p*
*Abemus chloropterus*					
No. squares visited	Urbanization level	−0.2114± 0.1895	1.2438	1	0.2647
	Sex	0.0766 ± 0.1628	0.2213	1	0.6381
	Urbanization level × Sex	−0.0689 ± 0.1938	0.1264	1	0.7222
No. inner squares visited	Urbanization level	0.0477 ± 0.2059	0.0538	1	0.8166
	Sex	0.3478 ± 0.1931	3.2445	1	0.0717
	Urbanization level × Sex	−0.2927 ± 0.2287	1.6383	1	0.2006
Time to wall, s	Urbanization level	−0.0192 ± 0.2065	0.0087	1	0.9258
	Sex	−0.1047 ± 0.2002	0.2735	1	0.6010
	Urbanization level × Sex	−0.1138 ± 0.2394	0.2261	1	0.6345
Escape duration, s	Urbanization level	−0.0697 ± 0.1137	0.3761	1	0.5397
	Sex	0.0213 ± 0.0993	0.0459	1	0.8304
	Urbanization level × Sex	−0.0359 ± 0.1177	0.0930	1	0.7604
Escape distance, no. segments	Urbanization level	−0.3215 ± 0.2792	1.3254	1	0.2496
	Sex	−0.0775 ± 0.2213	0.1226	1	0.7263
	Urbanization level × Sex	0.0116 ± 0.2721	0.0018	1	0.9659
*Ocypus nitens*					
No. squares visited	Urbanization level	−0.7211 ± 0.4642	2.4129	1	0.1203
	Sex	0.5603 ± 0.1934	8.3955	1	0.0038
	Urbanization level × Sex	0.3110 ± 0.3911	0.6323	1	0.4265
No. inner squares visited	Urbanization level	−0.3355 ± 0.3120	1.1562	1	0.2822
	Sex	0.1008 ± 0.1628	0.3830	1	0.5360
	Urbanization level × Sex	0.3740 ± 0.3344	1.2504	1	0.2635
Time to wall, s	Urbanization level	0.4853 ± 0.3343	2.2398	2	0.3263
	Sex	−0.3049 ± 0.1891	2.5991	1	0.1069
	Urbanization level × Sex	−0.6724 ± 0.3636	3.4190	1	0.0644
Escape duration, s	Urbanization level	−0.0721 ± 0.1425	0.2561	1	0.6128
	Sex	0.0236 ± 0.0849	0.0773	1	0.7810
	Urbanization level × Sex	0.1457 ± 0.1552	0.8824	1	0.3475
Escape distance, no. segments	Urbanization level	−0.1644 ± 0.4079	0.1624	1	0.6870
	Sex	0.0817 ± 0.2340	0.1218	1	0.7271
	Urbanization level × Sex	0.2663 ± 0.4376	0.3704	1	0.5428
*Platydracus fulvipes*					
No. squares visited	Urbanization level	−0.6457 ± 0.5083	1.6137	1	0.2040
	Sex	0.1687 ± 0.2800	0.3628	1	0.5469
	Urbanization level × Sex	0.7405 ± 0.4737	2.4436	1	0.1180
No. inner squares visited	Urbanization level	−0.2783 ± 0.3037	0.8398	1	0.3595
	Sex	−0.1170 ± 0.1879	0.3877	1	0.5335
	Urbanization level × Sex	0.3035 ± 0.3224	0.8860	1	0.3466
Time to wall, s	Urbanization level	0.0525 ± 0.2071	0.0643	1	0.7998
	Sex	−0.0281 ± 0.1064	0.0696	1	0.7919
	Urbanization level × Sex	−0.1364 ± 0.1680	0.6592	1	0.4168
Escape duration, s	Urbanization level	−0.0250 ± 0.0946	0.0697	1	0.7917
	Sex	−0.0019 ± 0.0638	0.0009	1	0.9759
	Urbanization level × Sex	0.0456 ± 0.1028	0.1964	1	0.6576
Escape distance, no. segments	Urbanization level	0.2747 ± 0.2750	0.9975	1	0.3179
	Sex	0.2554 ± 0.1951	1.7147	1	0.1904
	Urbanization level × Sex	−0.2015 ± 0.2977	0.4582	1	0.4984

## Data Availability

The data presented in this study are available on request from the corresponding author.

## Supplementary Materials

The following supporting information can be downloaded at: https://www.mdpi.com/article/10.3390/insects13080757/s1, Figure S1: Silhouette plots to identify possible groupings of the behavioral measures for *Abemus chloropterus* (A), *Ocypus nitens* (B) and *Platydracus fulvipes* (C); Table S1: Spearman correlations between the tested behavioral measures (average of the two trials for each measure) in the three tested rove beetle species collected in rural and urban habitats. Values in bold denote significant (*p* < 0.05) correlations; Table S2: Mean ± SE values of the studied behavioral measures of the rural and urban rove beetles.
