# Supplementary material for: Urban Individuals of Three Rove Beetle Species Are Not More Exploratory or Risk-Taking Than Rural Conspecifics
Source: Insects. 2022 Aug 22;13(8):757. doi: 10.3390/insects13080757 (PMC9409932; doi:10.3390/insects13080757)
Supplement: Supplementary file 1 [file insects-13-00757-s001.zip › insects-1802603-supplementary.pdf]

*Supplementary material to the article*

# Urban individuals of three rove beetle species are not more exploratory or risk-taking than rural conspecifics

**by Tibor Magura, Roland Horváth, Szabolcs Mizser, Mária Tóth, Dávid D. Nagy Réka Csicsek, Emőke Balla and Gábor L. Lövei**

**Figure S1.** Silhouette plots to identify possible groupings of the behavioural measures for *Abemus chloropterus* (A), *Ocypus nitens* (B) and *Platydracus fulvipes* (C).

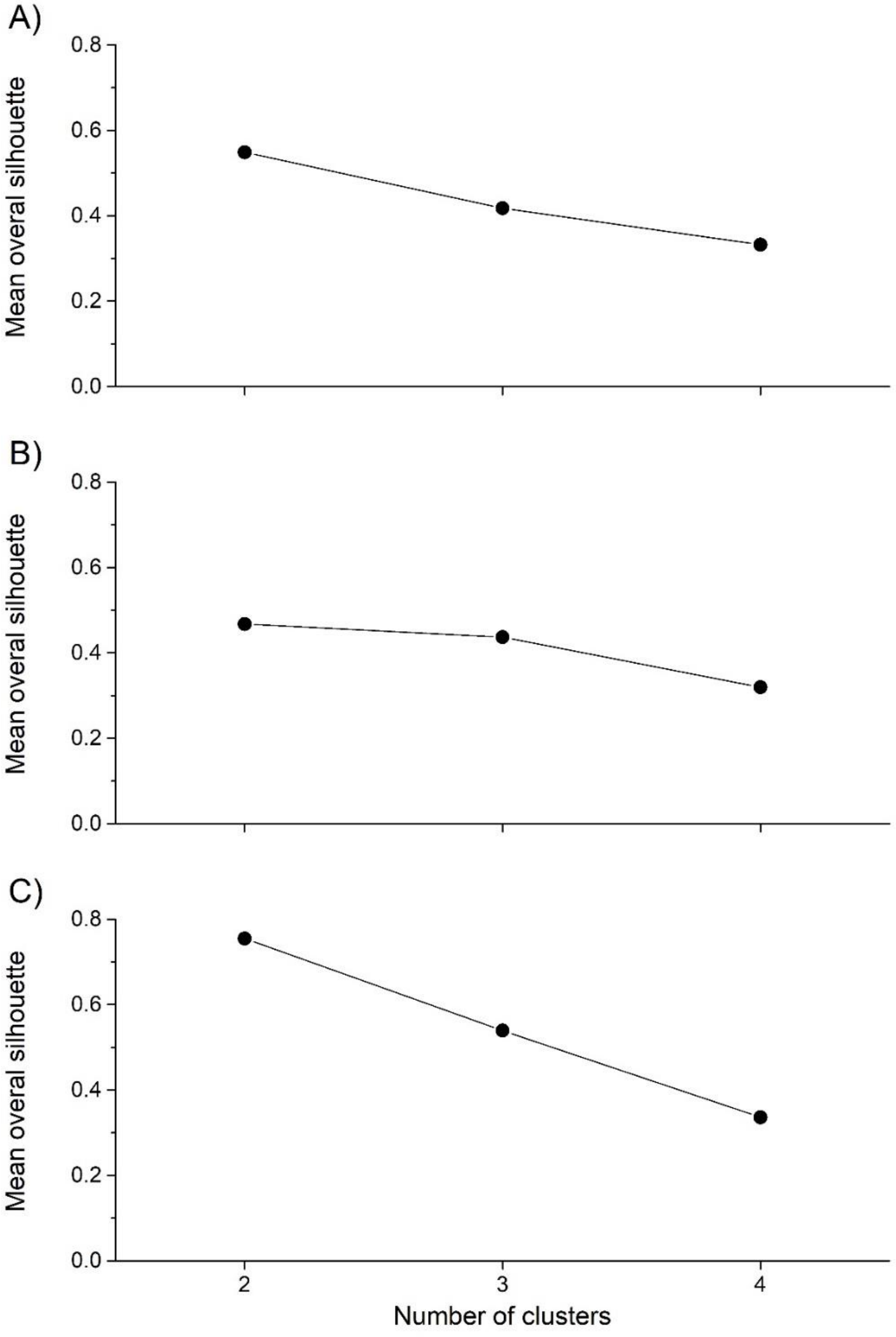

**Table S1.** Spearman correlations between the tested behavioural measures (average of the two trials for each measure) in the three tested rove beetle species collected in rural and urban habitats. Values in bold denote significant ($p$<0.05) correlations.

| ***Abemus chloropterus*** | **No. squares visited** | **No. inner squares visited** | **Time to wall** | **Escape duration** | **Escape distance** |
|---|---|---|---|---|---|
| No. squares visited | 1.0000 | **0.3634** | **-0.4585** | -0.0039 | -0.0153 |
| No. inner squares visited | **0.3634** | 1.0000 | 0.1915 | -0.1582 | -0.1036 |
| Time to wall | **-0.4585** | 0.1915 | 1.0000 | **-0.2473** | -0.1216 |
| Escape duration | -0.0039 | -0.1582 | **-0.2473** | 1.0000 | **0.8621** |
| Escape distance | -0.0153 | -0.1036 | -0.1216 | **0.8621** | 1.0000 |

| *Ocypus nitens* | No. squares visited | Inner square visit | Time to wall | Escape duration | Escape distance |
|---|---|---|---|---|---|
| No. squares visited | 1.0000 | **0.5251** | **-0.3492** | 0.2239 | **0.3375** |
| No. inner squares visited | **0.5251** | 1.0000 | -0.1670 | -0.0576 | 0.0136 |
| Time to wall | **-0.3492** | -0.1670 | 1.0000 | **-0.2831** | **-0.3733** |
| Escape duration | 0.2239 | -0.0576 | **-0.2831** | 1.0000 | **0.8839** |
| Escape distance | **0.3375** | 0.0136 | **0.3733** | **0.8839** | 1.0000 |

| *Platydracus fulvipes* | No. squares visited | Inner square visit | Time to wall | Escape duration | Escape distance |
|---|---|---|---|---|---|
| No. squares visited | 1.0000 | **0.6910** | **-0.8244** | 0.1706 | 0.1455 |
| No. inner squares visited | **0.6910** | 1.0000 | **-0.5375** | 0.1135 | 0.0065 |
| Time to wall | **-0.8244** | **-0.5375** | 1.0000 | -0.1431 | -0.1005 |
| Escape duration | 0.1706 | 0.1135 | -0.1431 | 1.0000 | **0.8340** |
| Escape distance | 0.1455 | 0.0065 | -0.1005 | **0.8340** | 1.0000 |

**Table S2.** Mean ± SE values of the studied behavioural measures of the rural and urban rove beetles.

| | **No. squares visited** | **No. inner squares visited** | **Time to wall** | **Escape duration** | **Escape distance** |
|---|---|---|---|---|---|
| *Abemus chloropterus* | | | | | |
| Rural females | 19.00 ± 3.78 | 4.17 ± 0.73 | 20.95 ± 9.33 | 4.89 ± 3.12 | 4.75 ± 1.56 |
| Rural males | 19.13 ± 1.67 | 6.11 ± 0.64 | 9.68 ± 2.45 | 3.30 ± 0.91 | 3.71 ± 0.50 |
| Urban females | 15.60 ± 1.57 | 4.53 ± 0.47 | 14.11 ± 4.08 | 1.90 ± 0.73 | 2.90 ± 0.47 |
| Urban males | 15.25 ± 0.89 | 4.79 ± 0.32 | 22.89 ± 3.50 | 1.73 ± 0.41 | 2.78 ± 0.27 |
| *Ocypus nitens* | | | | | |
| Rural females | 22.71 ± 4.04 | 5.25 ± 0.81 | 30.03 ± 7.44 | 1.93 ± 0.67 | 3.42 ± 0.59 |
| Rural males | 29.55 ± 2.39 | 5.65 ± 0.51 | 9.28 ± 2.65 | 1.97 ± 0.49 | 4.02 ± 0.52 |
| Urban females | 11.67 ± 3.72 | 3.67 ± 1.05 | 35.70 ± 17.18 | 1.02 ± 0.46 | 3.00 ± 1.24 |
| Urban males | 29.60 ± 2.07 | 5.95 ± 0.43 | 12.36 ± 2.06 | 3.00 ± 0.68 | 4.46 ± 0.49 |
| *Platydracus fulvipes* | | | | | |
| Rural females | 11.95 ± 2.69 | 4.35 ± 0.64 | 36.78 ± 7.66 | 1.11 ± 0.33 | 2.85 ± 0.41 |
| Rural males | 16.42 ± 1.93 | 3.98 ± 0.41 | 37.93 ± 4.63 | 1.95 ± 0.65 | 3.80 ± 0.43 |
| Urban females | 5.90 ± 1.60 | 3.20 ± 0.61 | 51.51 ± 13.14 | 1.30 ± 0.45 | 4.60 ± 1.37 |
| Urban males | 16.56 ± 2.00 | 4.06 ± 0.38 | 37.50 ± 4.30 | 1.93 ± 0.39 | 4.50 ± 0.42 |
